# A novel activation function based recurrent neural networks and their applications on sentiment classification and dynamic problems solving

**DOI:** 10.3389/fnbot.2022.1022887

**Published:** 2022-09-23

**Authors:** Qingyi Zhu, Mingtao Tan

**Affiliations:** ^1^School of Electronics and Internet of Things, Sichuan Vocational College of Information Technology, Guangyuan, China; ^2^School of Computer and Electrical Engineering, Hunan University of Arts and Science, Changde, China

**Keywords:** non-linear activation function, recurrent neural networks, sentiment classification, dynamic sylvester equation, robot manipulator

## Abstract

In this paper, a nonlinear activation function (NAF) is proposed to constructed three recurrent neural network (RNN) models (Simple RNN (SRNN) model, Long Short-term Memory (LSTM) model and Gated Recurrent Unit (GRU) model) for sentiment classification. The Internet Movie Database (IMDB) sentiment classification experiment results demonstrate that the three RNN models using the NAF achieve better accuracy and lower loss values compared with other commonly used activation functions (AF), such as ReLU, SELU etc. Moreover, in terms of dynamic problems solving, a fixed-time convergent recurrent neural network (FTCRNN) model with the NAF is constructed. Additionally, the fixed-time convergence property of the FTCRNN model is strictly validated and the upper bound convergence time formula of the FTCRNN model is obtained. Furthermore, the numerical simulation results of dynamic Sylvester equation (DSE) solving using the FTCRNN model indicate that the neural state solutions of the FTCRNN model quickly converge to the theoretical solutions of DSE problems whether there are noises or not. Ultimately, the FTCRNN model is also utilized to realize trajectory tracking of robot manipulator and electric circuit currents computation for the further validation of its accurateness and robustness, and the corresponding results further validate its superior performance and widespread applicability.

## Introduction

Sentiment classification and time-varying problem solving are two typical problems in practical applications. Sentiment classification utilizes computational techniques and natural language processing to classify specific text into positive and negative categories (Abdi et al., 2018; Chen and Xie, 2020; Wang and Lin, 2020; Saeed et al., 2021). As the document information generated by users increasing rapidly, the analysis of this information becomes more and more important. Based on the analysis of this information, it can obtain the feedback information from students, products, services and events, which is useful for decision making of universities, companies and governments (Yaslan and Aldo, 2017). As a powerful tool for calculation and natural language processing, the recurrent neural network (RNN) is widely used in medical treatment (He et al., 2020), sentiment classification (Azadeh et al., 2017; Zablith and Osman, 2019) and other fields (Yu et al., 2019, 2022a,b; Wan et al., 2022a,b). Note that, the performance of the RNN models for sentiment classification depends on its activation function (AF), which implies that the selection of AF will affect the accuracy of sentiment classification. Therefore, several activation functions are designed, for example, Rectified Linear Unit (ReLU), Leaky Rectified Linear Unit (LReLU), Exponential Linear Unit (ELU) and Scaled ELU (SELU). Motivated by the above discussions, a non-linear activation function (NAF) is proposed in this work. The proposed NAF is applied to a two-layer Simple RNN (SRNN) model, a Long Short-term Memory (LSTM) model and a Gated Recurrent Unit (GRU) model for sentiment classification, respectively. For the purpose of comparison, the RNN models (SRNN, LSTM, and GRU) activated by other commonly used AFs are also used for the same sentiment classification task. The simulation results demonstrate that the three NAF-based RNN models have better accuracy and faster loss decline than the RNN models activated by other commonly used AFs.

On the other hand, solving time-varying problems is becoming increasingly important in the fields of science and engineering (Gong and Jin, 2021; Jin and Gong, 2021; Liu et al., 2021; Jin and Qiu, 2022; Jin et al., 2022a,b,c,d; Zhu et al., 2022). As most time-varying problems can be described by time-varying matrix equations, the RNN is also widely used to effectively solving them in the past few years (Zhang et al., 2002; Xiao and Zhang, 2014b; Xiao et al., 2019; Jin, 2021a; Liu et al., 2022). Zeroing neural network (ZNN) is a typical RNN model developing rapidly for time-varying matrix equations solving, and many researchers devote to improve the convergence and robustness of the ZNN model in recent years. For example, the varying-parameter ZNN model is proposed by Zhang et al. (2018a,b,c, 2020b,d). The varying-parameter ZNN model is a parallel processing approach with high-efficiency and high-precision, and its unique advantage is that it is a real-time solver without any pre-training. Moreover, a large number of relevant literatures reveal that the performances of ZNN model are intrinsically related to its AF. Therefore, researchers have designed many AFs such as LAF and PSAF, and the ZNN model activated by them could exponentially converge to the theoretical solutions of dynamic problems in ideal no noise environment (Zhang et al., 2018c). Besides, the SBPAF activated ZNN model even converges in finite time, and the RNZNN models in ref. (Li et al., 2013) achieve fixed-time convergence and strong robustness to noises. Furthermore, the varying-parameter ZNN model achieves super-exponential convergence and strong robustness by introducing a variable convergent factor γ (Zhang et al., 2020c; Xiao and He, 2021). Although the improvements of AF and convergent factor γ both lead to the improved performances of the ZNN model, this work focuses on the development of AF of the ZNN model, and a NAF is presented and employed to obtain a fixed-time convergent recurrent neural network (FTCRNN) model for further enhance its convergence and robustness, and the FTCRNN model not only has strong robustness to noises but also converges in fixed-time for time-varying problems solving.

Consequently, the main contributions and innovations of this work are summarized as follows:

(1) An NAF suits for different RNN models is proposed.
(2) Three NAF activated RNN models (SRNN, LSTM, and GRU) are developed for sentiment classifications.
(3) A robust and fixed-time convergent FTCRNN model for DSE problem solving and robot manipulator trajectory tracking is presented.
(4) An application of electric circuit currents calculation based on the FTCRNN model is designed for its further validation.

The rest of this paper is organized as follows. The introduction of the proposed NAF is presented in Section The proposed NAF. In Section NAF-Based RNN models for Sentiment classification, the sentiment classification problem is formulated and the experiments of sentiment classification are presented. In Section NAF-Based FTCRNN model for dynamic problems solving, the effectiveness and robustness of the NAF-based FTCRNN model are verified by mathematical analysis. Besides, the experiments of time-varying DSE problem solving, robot manipulator trajectory tracking and electric circuit currents calculation using the NAF-based FTCRNN model are provided. Ultimately, the conclusions and future research directions are discussed in Section Conclusions.

## The proposed NAF

AF is an important part of RNN, which has great influences on the performances of RNN models. For example, linear AF-based RNN models are suitable for linear problems solving, and non-linear AF-based RNN models can effectively solve the non-linear problems while the linear AF-based RNN models are powerless in non-linear problems.

The four basic AFs for RNN models to realize sentiment classification are presented in Table 1. The ReLU-based RNN models have the advantage of unnecessary pre-processing operation. However, ReLU is relatively sparse, the useful information of the ReLU-based RNN models is easy to be ignored. Therefore, various improved AFs are proposed [e.g., Leak ReLU (LReLU), Exponential Linear Unit (ELU), Scaled ELU (SELU)…].

**Table 1:** Recently reported AFs for sentiment classification.

**No**.	**AFs**	**Expression**
1	ReLU	ϕ(*x*) = *max*(0, *x*)
2	LReLU	$\phi (x)=\{\begin{array}{ll} x, & x>0\\ \alpha x, & otherwise \end{array}$
3	ELU	$\phi (x)=\{\begin{array}{ll} x, & x>0\\ \alpha \left(e^{x}-1\right), & otherwise \end{array}$
4	SELU	$\phi (x)=\{\begin{array}{ll} x, & x>0\\ \alpha e^{x}-\alpha , & otherwise \end{array}$
5	Tanh	$\phi \left(x\right)=\frac{e^{x}-e^{-x}}{e^{x}+e^{-x}}$

The convergence characteristics of RNN models are closely related to the slope of their AFs (Xiao et al., 2018). The adjustable slope AF based RNN models have better flexibility in various problems solving, and their slope can be set according to specific practical requirements. Obviously, when *x* > *0*, the slopes of AFs in Table 1 are constant and they are not adjustable. Therefore, in order to further improve the performance of the RNN models for sentiment classification and time-varying problems solving, and inspired by the method in Chen et al. (2020), a new NAF with flexible slope adjustment property is proposed.


(1) $$\begin{array}{l} \phi \left(x\right)=\alpha_{0}x+\alpha_{1}sign\left(x\right){\left|x\right|}^{2\phi_{1}-1}+\alpha_{2}sign\left(x\right){\left|x\right|}^{2\phi_{2}-1} \end{array}$$

where α_0_ > *0*, α_1_ > *0*, α_2_ > *0*, ϕ_1_ > *1, 0* < ϕ_2_ < *1*, and *sign (*•*)* is the signum function.

In order to select proper parameter values of NAF (1) for sentiment classification, the following two-step method is applied.

**Figure 1:**
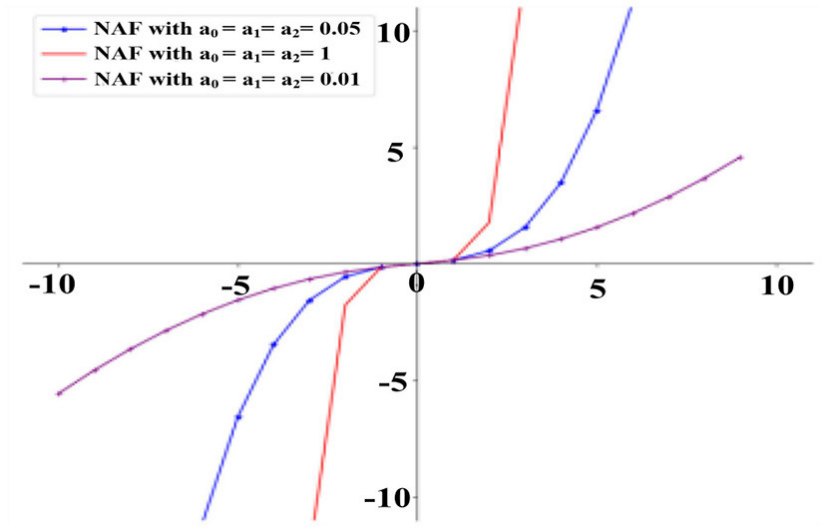
NAF (1) with different parameters.

Firstly, for the convenience of observation, the proposed NAF with different parameters (*a*_0_, α_1_*, and* α_2_) are plotted from the interval [−10, 10] in Figure 1. As seen in Figure 1, the slope of the NAF is closely related to the above parameters, and the middle blue curve with α_0_ = α_1_ = α_2_ = *0.05* is adopted.

**Figure 2:**
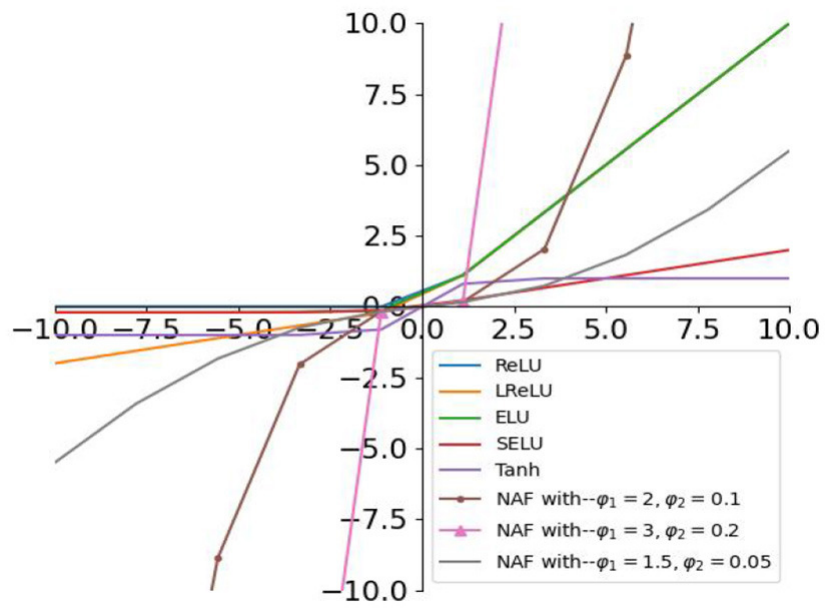
NAF (1) and AFs in Table 1.

Then, the AFs in Table 1 and the proposed NAF are plotted in Figure 2. As observed in Figure 2, the slope of the proposed NAF can also be adjusted by the parameters ϕ_1_ and ϕ_2_, and the middle curve with ϕ_1_ = *1.5*, ϕ_2_ = *0.1* is adopted.

Generally, the parameters in Equation (1) can be chosen arbitrarily as long as they satisfy α_0_ > *0*, α_1_ > *0*, α_2_ > *0*, ϕ_1_ > *1, 0* < ϕ_2_ < *1*. Here, we set α_0_ = α_1_ = α_2_ = *0.05*, ϕ_1_ = *1.5*, ϕ_2_ = *0.1* to ensure the NAF-based RNN models for sentiment classification achieve moderate convergence and robustness.

## NAF-based RNN models for sentiment classification

Sentiment classification is to judge whether the text information is positive or negative (e.g., good or bad, like or hate), which gathers classification information for users to make decision. In recent years, sentiment classification has been widely used in business, politics, social media, and other fields. In this section, three NAF activated RNN models (SRNN, LSTM and GRU) will be used for Internet Movie Database (IMDB) sentiment classification. Besides, the RNN models (SRNN, LSTM, and GRU) activated by other commonly used AFs in Table 1 are also used for IMDB sentiment classification for the purpose of comparison.

### Headings sentiment classification diagram

**Figure 3:**
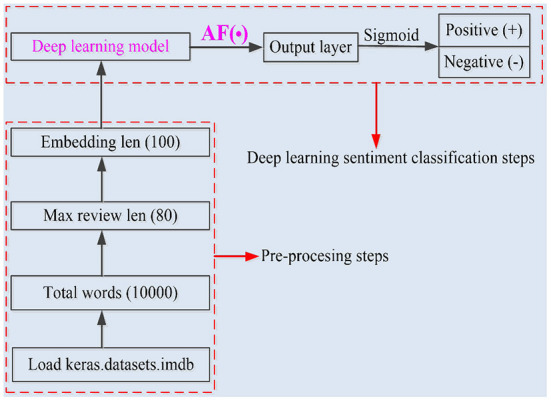
Sentiment classification diagram.

In this subsection, the diagram for IMDB sentiment classification is introduced. As shown in Figure 3, sentiment classification mainly includes the pre-processing part and the deep learning sentiment classification part.

The IMDB is used for sentiment analysis in this work, and the IMDB consists of 50,000 movie reviews. Before the training of the RNN models, the pre-processing steps are presented below.

Firstly, 25,000 reviews of the IMDB are used for test set, and the rest of 25,000 reviews are used for training set;

Secondly, the English vocabulary of the RNN models is set to be 10,000;

Thirdly, only 100 words of each comment can be read by the RNN models;

Finally, each English word is transformed to be a 100-dimensional vector.

After the pre-processing steps, the commonly used AF-based RNN models and the proposed NAF-based RNN models are used for sentiment classification, respectively.

It is worth pointing out that we use the command “word2vec” in Python to convert IMDB into vectors for calculation and training. “Word2vec” is an efficient model for training word vectors. It converts text data into low dimensional real number vectors through unsupervised training.

### Deep learning models for sentiment classification

The SRNN, LSTM and GRU models are used as deep learning models for sentiment classification in this work, and the corresponding introduction of the three RNN models are presented below.

#### SRNN model

**Figure 4:**
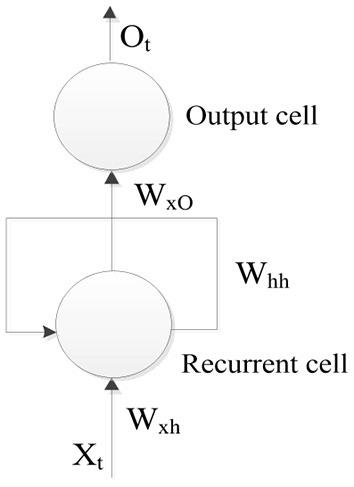
SRNN model.

The SRNN model is shown in Figure 4, and its operation can be expressed mathematically as


(2) $$\begin{array}{l} \begin{array}{c} H_{t}=f\left(W_{xh}X_{t}+W_{hh}H_{t-1}\right)\\ O_{t}=g\left(W_{xO}H_{t}\right) \end{array} \end{array}$$

where *X*_*t*_ is input at time. *H*_*t*_ is state at time. *W*_*xh*_*, W*_*hh*_ and *W*_*xO*_ are weight matrices.

#### LSTM model

**Figure 5:**
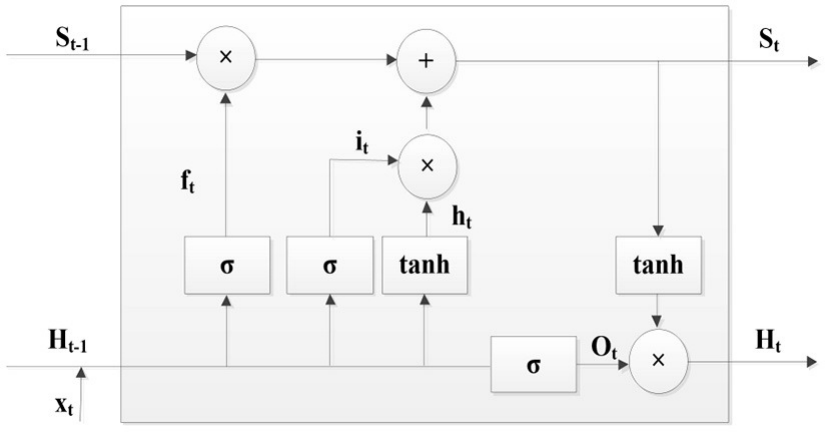
LSTM model.

Generally, the LSTM model consist of memory cell, input gate, output gate and forgetting gate, and the LSTM model is presented in Figure 5.

As a typical RNN model, the LSTM model has long-term or short-term memory, and the results of each LSTM cell are related to its current and past states. The LSTM model effectively solves the problems of gradient vanishing and gradient explosion, and its mathematical expression is presented below.


(3) $$\begin{array}{l} \begin{array}{c} f_{\text{t}}=\sigma \left(W_{f}\left(h_{t-1},x_{t}\right)+b_{f}\right)\;i_{t}=\sigma \left(W_{i}\left(h_{t-1},x_{t}\right)+b_{i}\right)\\ O_{\text{t}}=\sigma \left(W_{o}\left(h_{t-1},x_{t}\right)+b_{o}\right)\;h_{t}=\tanh\left(x_{t},H_{t-1}\right)\\ S_{t}=S_{t-1}f_{t}+h_{t}i_{t}\;H_{t}=\tanh\left(S_{t}\right)\times O_{t} \end{array} \end{array}$$

#### GRU model

The GRU model is the simplified version of the LSTM model, and it can also solve the problems of gradient vanishing and gradient explosion. Compared with the LSTM model, the GRU model only has the update gate and reset gate, and it is easier to be trained.

**Figure 6:**
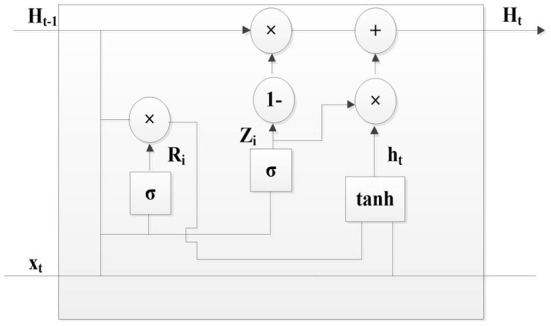
GRU model.

The GRU model is represented in Figure 6, and its mathematical expression is


(4) $$\begin{array}{l} \begin{array}{c} Z_{t}=\sigma \left(W_{z}\left(H_{t-1},x_{t}\right)\right)\;R_{t}=\sigma \left(W_{r}\left(H_{t-1},x_{t}\right)\right)\\ h_{t}=\tanh\left(R_{t}h_{t-1},x_{t}\right)\;H_{t}=\left(Z_{t}\times h_{t}\right)+\left(\left(1-Z_{t}\right)\times H_{t-1}\right) \end{array} \end{array}$$

### IMDB sentiment classification

In this subsection, the IMDB sentiment classification experiments are presented to verify the superior performances of the proposed NAF. The hardware and software of the computer used for sentiment classification are presented in Table 2. The three RNN models all use the categorical cross entropy loss function, and their training batch size values are 128 and 30 epochs. Moreover, the number of neurons in the SRNN, LSTM, and GRU models is set as 100 in the input layer, 64 in the hidden layer, 64 in the full connection layer and 1 in the output layer.

**Table 2:** Computer hardware and software.

CPU	Inter i5-7400
GPU	Inter HD Graphics 630
RAM	8 GB
Python version	3.0
tensorflow version	2.0

**Figure 7:**
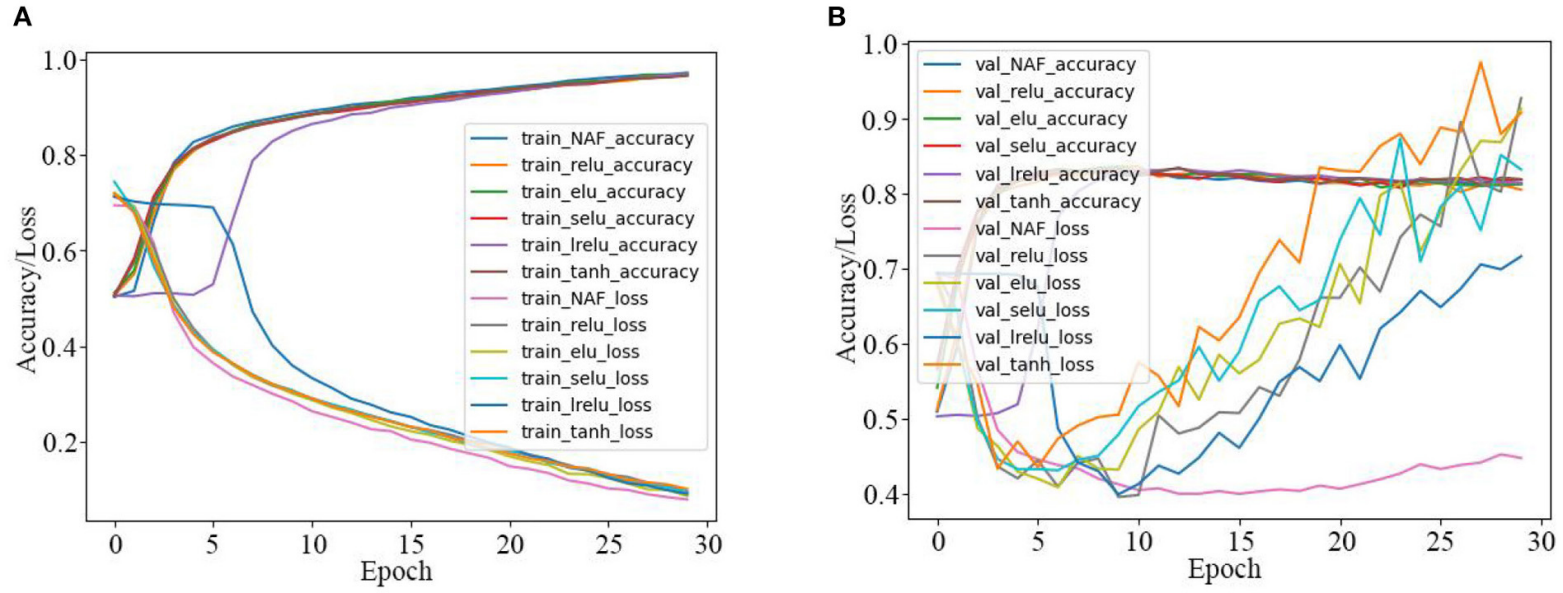
Training and test curves of SRNN model for IMDB sentiment classification. **(A)** Training curves. **(B)** Tested sentiment classification curves.

Firstly, the SRNN model activated by the proposed NAF and other recently reported AFs in Table 1 are used for the IMDB sentiment classification, and the experiment results are presented in Table 3 and Figure 7. As observed in Table 3, the NAF-based SRNN model and the SRNN model activated by other recently reported AFs all have good training effects with training accuracy >96.5% and training loss <0.93. However, the NAF-based SRNN model is the best one among them, and its training accuracy and training loss achieves 97.2700% and 0.0793, respectively. Moreover, we can also observe that the NAF-based SRNN model and the SRNN model activated by other recently reported AFs all have good sentiment classification effects with sentiment classification accuracy >83.62% and sentiment classification loss <0.9275. Furthermore, the sentiment classification accuracy of the NAF-based SRNN model even achieves 83.9000%, its loss value is 0.4473, which is the lowest of all the SRNN models.

**Table 3:** SRNN model with different AFs for IMDB sentiment classification.

**No**.	**AFs**	**Training accuracy (%)**	**Training loss**	**Val-accuracy (%)**	**Val-loss**
1	ReLU	96.7400	0.0949	83.6300	0.9275
2	LReLU	96.9000	0.0911	83.3200	0.7104
3	ELU	97.0000	0.0864	83.6200	0.9135
4	SELU	96.6600	0.0957	83.0600	0.8323
5	Tanh	96.5700	0.1010	83.4700	0.9083
6	NAF	97.2700	0.0793	83.9000	0.4473

In order to further clearly illustrate the training and sentiment classification effects of the SRNN model activated by the five AFs, the data in Table 3 is plotted in Figure 7. Figures 7A,B are the training curves and the tested sentiment classification curves, respectively. The X-axis represents the number of iterations, and the Y-axis represents the accuracy and loss values. As observed in Figure 7A, the training accuracy of the NAF-based SRNN model is clearly higher than other SRNN models, and the training loss value is always smaller than other SRNN models. In addition, as observed in Figure 7B, the test loss value of the NAF-based SRNN model is extraordinary stable comparing with other SRNN models.

**Figure 8:**
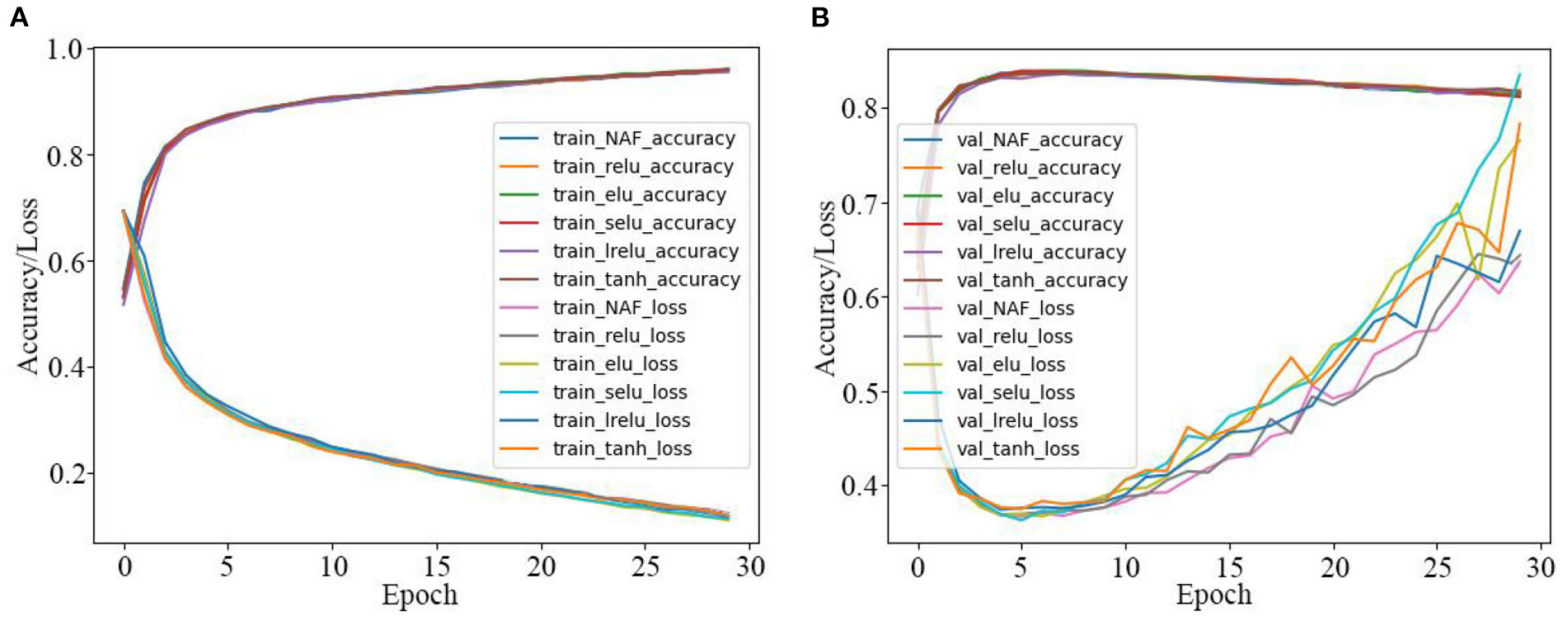
Training and test curves of LSTM model for IMDB sentiment classification. **(A)** Training curves. **(B)** Tested sentiment classification curves.

Secondly, the LSTM model activated by the proposed NAF and other recently reported AFs in Table 1 are also used for the IMDB sentiment classification, and the corresponding experiment results are presented in Table 4 and Figure 8. From Table 4 and Figure 8, it is clear that the sentiment classification accuracy of the LSTM model activated by the proposed NAF achieves 83.8700%, and its loss value is also the best one among them.

**Table 4:** LSTM model with different AFs for IMDB sentiment classification.

**No**.	**AFs**	**Train accuracy (%)**	**Train loss**	**Val-accuracy (%)**	**Val-loss**
1	ReLU	95.5400	0.1227	83.6900	0.6315
2	LReLU	95.6000	0.1222	83.4200	0.6693
3	ELU	96.0300	0.1103	83.8900	0.7652
4	SELU	95.9400	0.1137	83.8900	0.8349
5	Tanh	95.8100	0.1189	83.6800	0.7827
6	NAF	95.7600	0.1181	83.8700	0.6226

**Figure 9:**
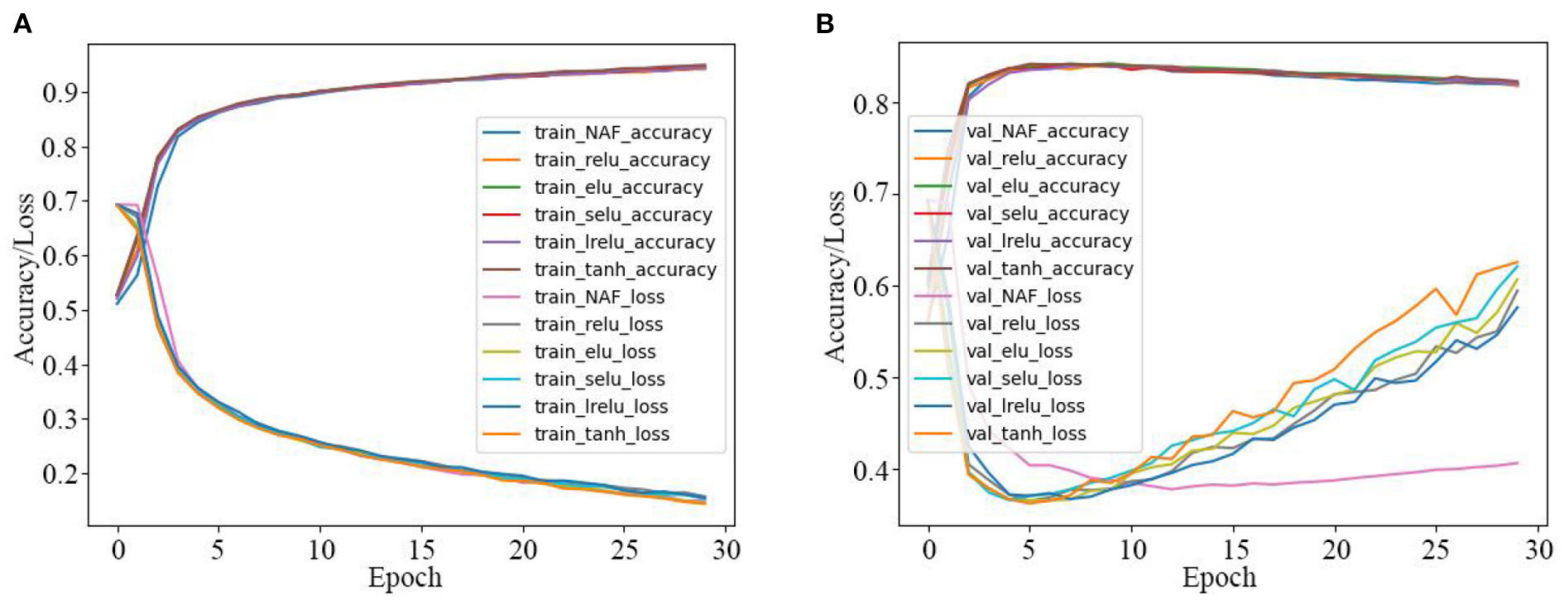
Training and test curves of GRU model for IMDB sentiment classification. **(A)** Training curves. **(B)** Tested sentiment classification curves.

Thirdly, the IMDB sentiment classification is realized by the GRU model activated by the proposed NAF and other Afs in Table 1, and the corresponding results are presented in Table 5 and Figure 9. As seen in Table 5 and Figure 9, the sentiment classification loss value of the GRU model activated by the proposed NAF is the lowest than other models, and it achieves best sentiment classification accuracy with fewer epochs among all the models.

**Table 5:** GRU model with different Afs for IMDB sentiment classification.

**No**	**AFs**	**Train accuracy (%)**	**Train loss**	**Val-accuracy (%)**	**Val-loss**
1	ReLU	94.2500	0.1575	83.8900	0.5937
2	LReLU	94.4600	0.1553	84.0200	0.5755
3	ELU	94.7400	0.1475	84.1800	0.6061
4	SELU	94.5200	0.1533	84.0100	0.6202
5	Tanh	94.8000	0.1442	84.1300	0.6249
6	NAF	94.8100	0.1458	84.1900	0.4056

**Figure 10:**
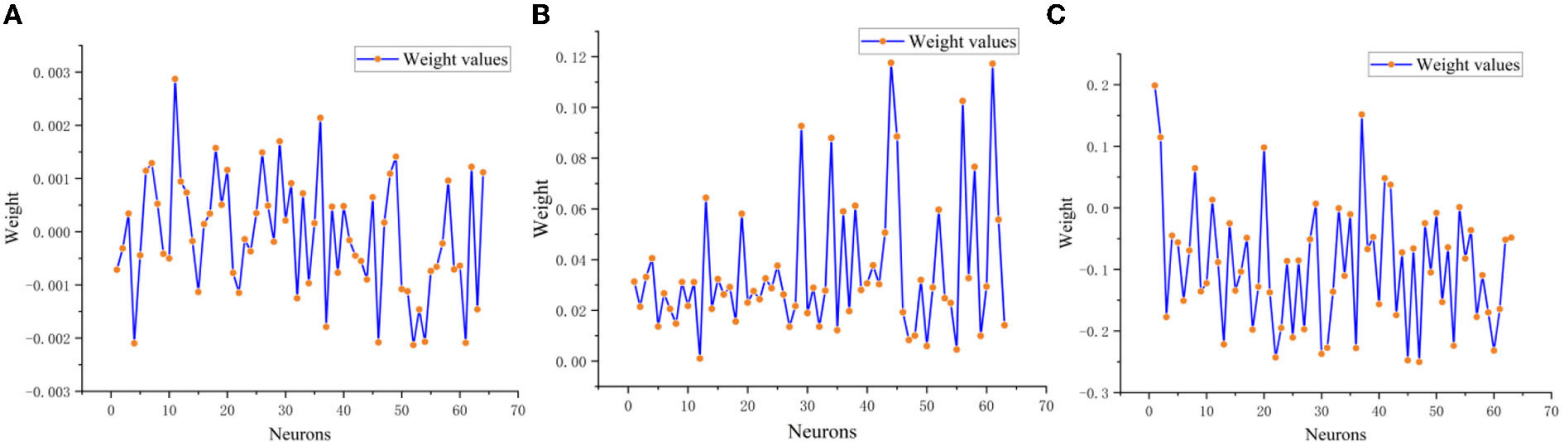
Output layer weights of SRNN, LSTM, and GRU model. **(A)** SRNN output layer weights. **(B)** LSTM output layer weights. **(C)** GRU output layer weights.

Finally, in order to illustrate the training process of SRNN, LSTM and GRU models for sentiment classification more clearly, the output layer weights of three models are presented in Figure 10.

## NAF-based FTCRNN model for dynamic problems solving

Sylvester equation is frequently applied in science and engineering fields, and many problems can be solved by finding the solution of Sylvester equations (Darouach, 2006; Zhang et al., 2018c).

In this section, for the purpose of verifying the superior performance of the FTCRNN model, preliminary mathematical preparation and theoretical analysis are given. Besides, the NAF-based FTCRNN model is used for three practical dynamic problems solving, which are dynamic Sylvester equation solving, robot manipulator trajectory tracking and electric circuit currents calculation. Moreover, the original ZNN activated by other recently reported AFs in Table 1 are also used to solve the above three practical dynamic problems for the purpose of comparison.

### The dynamic sylvester equation

The DSE problem can be summarized in the following matrix equation.


(5) $$\begin{array}{l} A\left(t\right)X\left(t\right)-X\left(t\right)B\left(t\right)=-C\left(t\right)\in {\mathbb{R}}^{n\times n} \end{array}$$

where *t* represents time, *X(t)* ∈ ℝ^*n*×*n*^ is the unknown matrix to be solved. *A(t)* ∈ ℝ^*n*×*n*^, *B(t)* ∈ ℝ^*n*×*n*^ and C*(t)* ∈ ℝ^*n*×*n*^ are the known smooth dynamic coefficient matrices, and their time derivatives matrices are also assumed to be known.

### ZNN model

As a special type of RNN, the ZNN model plays an important role in solving time-varying problems in the past few years. Following the methods in Zhang et al. (2002), the ZNN model for solving DSE problems can be constructed as below.

Firstly, according to Equation (5), a dynamic error function *E(t)* is adopted.


(6) $$\begin{array}{l} E\left(t\right)=A\left(t\right)X\left(t\right)-X\left(t\right)B\left(t\right)+C\left(t\right) \end{array}$$

where *E*(*t*) ∈ ℝ^*n*×*n*^ is the error matrix, and its time derivative *dE*(*t*)/*dt* should be negative-definite to ensure each element of *E(t)* converging to 0.

Secondly, the following formula is adopted for the convergence of *E(t)*.


(7) $$\begin{array}{l} \frac{dE\left(t\right)}{dt}=-\gamma \text{Ψ}\left(E\left(t\right)\right) \end{array}$$

where γ > 0 is an adjustable parameter related to the convergence performance of the ZNN model. ψ(•):ℝ^*n*×*n*^ → ℝ^*n*×*n*^ is an nonlinear AF array, and ϕ *(*•*)* is the element of the matrix ψ*(*•*)*.

The time differential of equation (6) is


(8) $$\begin{array}{l} \frac{dE\left(t\right)}{dt}=\overset{•}{A}\left(t\right)X\left(t\right)+A\left(t\right)\overset{•}{X}\left(t\right)-\overset{•}{X}\left(t\right)B\left(t\right)-X\left(t\right)\overset{•}{B}\left(t\right)\\ \;+\overset{•}{C}\left(t\right) \end{array}$$

At last, substituting equation (7) into (8), the ZNN model for solving DSE is realized.


(9) $$\begin{array}{l} A\left(t\right)\overset{•}{X}\left(t\right)-\overset{•}{X}\left(t\right)B\left(t\right)=-\overset{•}{A}\left(t\right)X\left(t\right)+X\left(t\right)\overset{•}{B}\left(t\right)-\overset{•}{C}\left(t\right)\\ \;-\gamma \text{Ψ}\left(E\left(t\right)\right) \end{array}$$

ZNN model (9) is stable as long as AF ψ(•) is a monotonically increasing function (Zhang et al., 2007, 2008), and the recently reported AFs are listed in Table 6.

**Table 6:** Recently reported AFs for ZNN model (9).

**No**.	**AFs**	**Expression**
1	Linear activation function (LAF)	ϕ(*x*) = *x*
2	Power-Sigmoid activation function (PSAF)	$\phi (x=)=\{\begin{array}{ll} x^{p}, & \left|x\right|\geq 1\\ \frac{1+e^{-\xi }-e^{-\xi x}}{1+e^{-\xi }+e^{-\xi x}}, & otherwise \end{array}$
3	Sign-bi-power activation function (SBPAF)	$\phi \left(x\right)=\frac{1}{2}sgn^{\xi_{1}}\left(x\right)+\frac{1}{2}sgn^{\xi_{2}}\left(x\right)$
4	Versatile activation function (VAF)	$\phi \left(x\right)=\frac{b_{1}}{p}\exp\left(|x{|}^{p}\right)|x{|}^{1-p}\text{sgn}x+b_{2}x+b_{3}\text{sgn}\left(x\right)$

### The NAF-based FTCRNN model

The FTCRNN model is obtained by introducing the NAF (1) to the ZNN model (9), and its expression is


(10) $$\begin{array}{l} \overset{•}{E}(t)=-\gamma [\alpha_{0}(E(t))+\alpha_{1}sign(E(t))\left|E^{2\phi_{1}-1}(t)\right|\\ \;+\alpha_{2}sign(E(t))\left|E^{2\phi_{2}-1}(t)\right|] \end{array}$$

Then, the FTCRNN model for solving DSE is shown as below.


(11) $$\begin{array}{l} A\left(t\right)\overset{•}{X}\left(t\right)-\overset{•}{X}\left(t\right)B\left(t\right)=-\overset{•}{A}\left(t\right)X\left(t\right)+X\left(t\right)\overset{•}{B}\left(t\right)-\overset{•}{C}\left(t\right)\\ \;+\overset{•}{E}\left(t\right) \end{array}$$

To further analyze its robustness, the FTCRNN model with additive noise *N(t)* is presented as follows.


(12) $$\begin{array}{l} A\left(t\right)\overset{•}{X}\left(t\right)-\overset{•}{X}\left(t\right)B\left(t\right)=-\overset{•}{A}\left(t\right)X\left(t\right)+X\left(t\right)\overset{•}{B}\left(t\right)-\overset{•}{C}\left(t\right)+\overset{•}{E}\left(t\right)\\ \;+N\left(t\right) \end{array}$$

**Figure 11:**
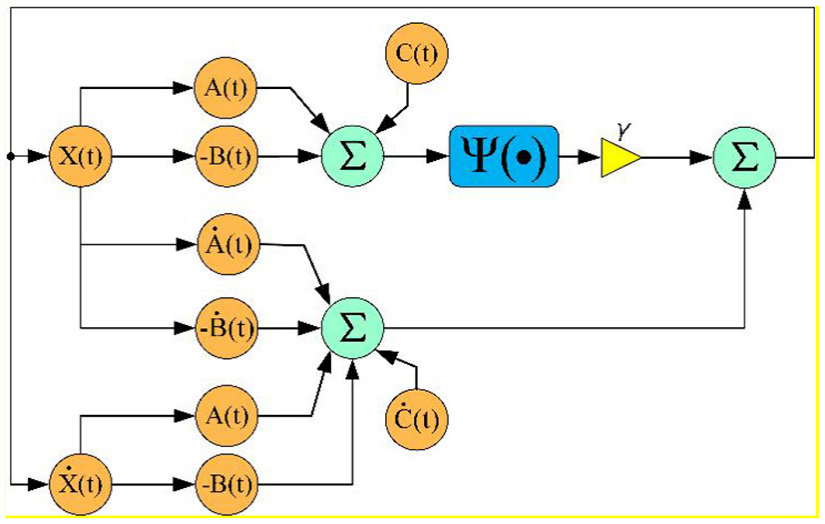
FTCRNN model algorithm diagram.

The corresponding algorithm diagram of the FTCRNN model for solving TODSE problem is presented in Figure 11. *A(t), B(t), C(t), X(t)*, and ψ*(*•*)* are the previous defined matrices. Each orange circle represents a time-varying matrix or their derivative matrix, and the number of neurons in each orange circle depends on the number of matrix elements. Besides, the convergence factor γ is set as 1 for fair comparison.

### FTCRNN analysis

In order to verify the fixed-time convergence and robustness to noise of the proposed FTCRNN model, the following Lemma 1 is presented in advance.

**Lemma 1** (Chen et al., **2020****)**. If *V(*•*): R*^*n*^ → *R* + ⋃{0} is a continuous radially unbounded function, and the following two conditions hold.

(I) *V(e(t))* = 0 **⇔***e(t)* = 0;

(II) Any *e(t)* in system (13) satisfies


(13) $$\begin{array}{l} \overset{•}{V}\left(e\left(t\right)\right)\leq -\alpha V^{\xi }\left(e\left(t\right)\right)-\beta V^{\eta }\left(e\left(t\right)\right)-\delta V\left(e\left(t\right)\right),\\ t\in \left[0,+\infty \right] \end{array}$$

where α, β, δ > *0, 0* < ξ < *1*, and η > *1*. Then the dynamic system (13) is fixed-time stability, and


(14) $$\begin{array}{l} T_{m}=\frac{1}{\delta \left(1-\xi \right)}\ln\;\left(1+\frac{\delta }{\alpha }\right)+\frac{1}{\delta \left(\eta -1\right)}\ln\;\left(1+\frac{\delta }{\beta }\right) \end{array}$$

**Definition 1** (Chen et al., 2020). The dynamic system (13) achieves finite-time stability, if there exists a constant *T(e(0))* > *0* such that $\underset{t\to T\left(e\left(0\right)\right)}{\lim}||e\left(t\right)|{|}_{1}$ and ||*e(t)*||_1_ = *0* for ∀*t* > *T (e(0))*, where *T(e(0))* is the settling time.

**Definition 2** (Chen et al., 2020). The dynamic system (13) achieves fixed-time stability, if two conditions are satisfied: (i) The dynamic system (13) achieves finite-time stability; (ii) For any *e(0)*, there exists a fixed constant *T*_*m*_ > *0* such that *T(e(0))* ≤ *T*_*m*_.

***Proof:*** Let *U(s)* = *V*^1−ξ^*(s)*, then we have


(15) $$\begin{array}{l} \overset{•}{V}\left(s\right)=\frac{1}{1-\xi }\overset{•}{U}\left(s\right)V^{\xi }\left(s\right) \end{array}$$

Combining Equations (13, 15) yields


(16) $$\begin{array}{l} \frac{1}{1-\xi }\overset{•}{U}(e(t))V^{\xi }(e(t))\leq -\alpha V^{\xi }(e(t))-\beta V^{\eta }(e(t))\\ \;-\delta V(e(t)) \end{array}$$

Then, we can rewrite Equation (16) as


(17) $$\begin{array}{l} \overset{•}{U}\left(e\left(t\right)\right)\leq -\alpha \left(1-\xi \right) \end{array}$$

Because $\overset{•}{U}\left(e\left(t\right)\right)\leq -\alpha \left(1-\xi \right)$, there always exists a constant $T\left(e\left(0\right)\right)=\frac{U\left(e\left(0\right)\right)}{\alpha \left(1-\zeta \right)}>0$, such that $\underset{t\to Te\left(\left(0\right)\right)}{\lim}U(e(t))=0$ and *U*(*e*(*t*)) = 0 for ∀*t* > *T*(*e*(0)). In view of *U*(*e*(0)) = 0⇔*V*(*e*(*t*)) = 0⇔*e*(*t*) = 0, so there exists a constant *T*(*e*(0)) > 0 such that $\underset{t\to T\left(e\left(0\right)\right)}{\lim}||e\left(t\right)|{|}_{1}=0$ and ||*e*(*t*)||_1_ = 0 for ∀*t* > *T*(*e*(0)). According to Definition 1, the dynamic system (13) is finite-time stable.

Equation (16) can be written in the following form


(18) $$\begin{array}{l} \frac{dt}{dV\left(e\left(t\right)\right)}\geq \frac{1}{-\alpha V^{\xi }\left(e\left(t\right)\right)-\beta V^{\eta }\left(e\left(t\right)\right)-\delta V\left(e\left(t\right)\right)} \end{array}$$

and we have


(19) $$\begin{array}{l} T\left(e\left(0\right)\right)\leq \int_{0}^{V\left(e\left(0\right)\right)}\frac{1}{\alpha s^{\xi }+\beta s^{\eta }+\delta s}ds \end{array}$$

Then, the analysis of Equation (19) should be divided into the following two cases.

(i) If 0 ≤ *V*(*e*(0)) ≤ 1,


(20) $$\begin{array}{l} T\left(e\left(0\right)\right)\leq \int_{0}^{1}\frac{1}{\alpha {\text{s}}^{\xi }+\beta s^{\eta }+\delta s}ds\leq \int_{0}^{1}\frac{1}{\alpha {\text{s}}^{\xi }+\delta s}ds \end{array}$$

Let ω = *s*^1−ξ^, we have *dω* = (1 − ζ)*s*^−ξ^*ds*, thus


(21) $$\begin{array}{l} \int_{0}^{1}\frac{1}{\alpha {\text{s}}^{\xi }+\delta s}ds=\frac{1}{1-\xi }\int_{0}^{1}\frac{s^{\xi }}{\alpha s^{\xi }+\delta s}d\omega \\ \;=\frac{1}{\delta \left(1-\xi \right)}\ln\;\left(1+\frac{\delta }{\alpha }\right) \end{array}$$

Therefore, we can obtain


(22) $$\begin{array}{l} T\left(e\left(0\right)\right)\leq \frac{1}{\delta \left(1-\xi \right)}\ln\;\left(1+\frac{\delta }{\alpha }\right) \end{array}$$

(ii) If *V*(*e*(0)) ≤ 1,


(23) $$\begin{array}{l} T\left(e\left(0\right)\right)\leq \int_{0}^{1}\frac{1}{\alpha s^{\xi }+\beta s^{\eta }+\delta s}ds+\int_{1}^{V\left(e\left(0\right)\right)}\frac{1}{\alpha s^{\xi }+\beta s^{\eta }+\delta s}ds\\ \;\leq \int_{0}^{1}\frac{1}{\alpha s^{\xi }+\delta s}ds+\int_{1}^{+\infty }\frac{1}{bs^{\eta }+\delta s}ds \end{array}$$

Let *c* = *s*^1−η^, we have *dc* = (1−η)*s*^−η^*ds*, thus


(24) $$\begin{array}{l} \int_{1}^{+\infty }\frac{1}{\beta {\text{s}}^{\eta }+\delta s}ds=\frac{1}{1-\eta }\int_{1}^{0}\frac{s^{\eta }}{\beta s^{\eta }+\delta s}dc\\ \;=\frac{1}{\delta \left(\eta -1\right)}\ln\;\left(1+\frac{\delta }{\beta }\right) \end{array}$$

Therefore, we can obtain


(25) $$\begin{array}{l} T\left(e\left(0\right)\right)\leq \frac{1}{\delta \left(\eta -1\right)}\ln\;\left(1+\frac{\delta }{\beta }\right) \end{array}$$

According to Definition 2, we can obtain that dynamic system (13) is stable in fixed-time *T*_*m*_.


(26) $$\begin{array}{l} T_{m}\leq \frac{1}{\delta \left(1-\xi \right)}\ln\;\left(1+\frac{\delta }{\alpha }\right)+\frac{1}{\delta \left(\eta -1\right)}\ln\;\left(1+\frac{\delta }{\beta }\right) \end{array}$$

The proof of Lemma 1 is completed.

According to Lemma 1, we will verify the fixed-time convergence and robustness to noises of the proposed FTCRNN model in the following two cases.

#### Case 1: Convergence analysis without noise

**Theorem 1**. If the solution of DSE (1) exists, the state solution *X(t)* of FTCRNN model (11) will converge to the theoretical solution X^*^(t) of DSE (1) in fixed-time *T*_*s*_.


$$\begin{array}{l} T_{s}\leq \frac{1}{2\alpha_{0}\left(1-\phi_{1}\right)}\ln\;\left(1+\frac{\alpha_{0}}{\alpha_{1}}\right)+\frac{1}{2\alpha_{0}\left(\phi_{2}-1\right)}\ln\;\left(1+\frac{\alpha_{0}}{\alpha_{2}}\right) \end{array}$$

***Proof***. According to Equation (10), the *ij*th dynamic error function *e*_*ij*_*(t)* of FTCRNN model (11) can be expressed as


(27) $$\begin{array}{l} {\overset{•}{e}}_{ij}(t)=-\gamma [\alpha_{0}(e_{ij}(t))+\alpha_{1}sign(e_{ij}(t))\left|e_{ij}{\;}^{2\phi_{1}-1}(t)\right|\\ \;+\alpha_{2}sign(e_{ij}(t))\left|e_{ij}{\;}^{2\phi_{2}-1}(t)\right|] \end{array}$$

When γ = *1*, we choose the following Lyapunov function.


(28) $$\begin{array}{l} V\left(e_{ij}\left(t\right)\right)=\frac{1}{2}{\left|e_{ij}\left(t\right)\right|}^{2} \end{array}$$

(29) $$\begin{array}{l} \overset{•}{V}(e_{ij}(t))=e_{ij}(t){\overset{•}{e}}_{ij}(t)=e_{ij}(t)\times \{-\gamma [\alpha_{0}(e_{ij}(t))\\ \;+\alpha_{1}sign(e_{ij}(t))\left|e_{ij}{\;}^{2\phi_{1}-1}(t)\right|\\ \;+\alpha_{2}sign(e_{ij}(t))\left|e_{ij}{\;}^{2\phi_{2}-1}(t)\right|]\}\\ \;=-\gamma \left(\alpha_{0}e_{ij}^{2}(t)+\alpha_{1}\left|e_{ij}{\;}^{2\phi_{1}}(t)\right|+\alpha_{2}\left|e_{ij}{\;}^{2\phi_{2}}(t)\right|\right)\\ \;=-(2\alpha_{0}V(e_{ij}(t))+2\alpha_{1}V^{\phi_{1}}(e_{ij}(t))\\ \;+2\alpha_{2}V^{\phi_{2}}(e_{ij}(t))) \end{array}$$

According to Lemma 1, the convergent time of FTCRNN model (11) can be obtained.


(30) $$\begin{array}{l} T_{ij}\leq \frac{1}{2\alpha_{0}\left(1-\phi_{1}\right)}\ln\;\left(1+\frac{\alpha_{0}}{\alpha_{1}}\right)+\frac{1}{2\alpha_{0}\left(\phi_{2}-1\right)}\ln\;\left(1+\frac{\alpha_{0}}{\alpha_{2}}\right) \end{array}$$

#### Case 2: Convergence analysis with noises

Noise interference is inevitable in the practical applications, therefore, it is necessary to consider the capability of anti-noises ability. Therefore, in this subsection, the robustness of the proposed FTCRNN model is analyzed, and the following theorem 2 guarantees the fixed-time stable of FTCRNN (12) in noisy environment.

**Theorem 2**. If FTCRNN model (12) is polluted by external noise *N(t)* with its *ij*th element satisfying |*n*_*ij*_(*t*)| ≤ γ|*e*_*ij*_(*t*)|, where σ ∈ *(0*, +∞*)*, |*e*_*ij*_*(t)*| and |*n*_*ij*_*(t)*| are the absolute values of the *ij*th element of *E(t)* and *N(t)*, respectively. In addition, suppose γ × min(α, α_1_, α_2_) > σ. Then, neural state solution *X(t)* of FTCRNN model (12) converges to the theoretical solution X^*^(t) of DSE (5) in fixed-time *T*_*s*_.


(31) $$\begin{array}{l} T_{s}\leq \frac{1}{\left(2\alpha_{0}+\sigma \right)\left(1-\phi_{1}\right)}\ln\;\left(1+\frac{2\alpha_{0}+\sigma }{2\alpha_{1}}\right)\\ \;+\frac{1}{\left(2\alpha_{0}+\sigma \right)\left(\phi_{2}-1\right)}\ln\;\left(1+\frac{2\alpha_{0}+\sigma }{2\alpha_{2}}\right) \end{array}$$

where the parameters *a*, α_1_, α_2_, ϕ_1_, ϕ_2_ are defined similar as before.

***Proof:*** The *ij*th dynamic error function *e*_*ij*_*(t)* of FTCRNN model (12) with noises can be expressed as


(32) $$\begin{array}{l} \overset{•}{e_{ij}}(t)=-\gamma [\alpha_{0}(e_{ij}(t))+\alpha_{1}sign(e_{ij}(t))\left|e_{ij}^{2\phi_{1}-1}(t)\right|\\ \;+\alpha_{2}sign(e_{ij}(t))\left|e_{ij}^{2\phi_{2}-1}(t)\right|]-n_{ij}(t) \end{array}$$

When γ = *1*, we choose the following Lyapunov function.


(33) $$\begin{array}{l} V\left(e_{ij}\left(t\right)\right)=\frac{1}{2}{\left|e_{ij}\left(t\right)\right|}^{2} \end{array}$$

(34) $$\begin{array}{l} \overset{•}{V}(e_{ij}(t))=e_{ij}(t){\overset{•}{e}}_{ij}(t)=e_{ij}(t)\times \{-\gamma [A(e_{ij}(t))\\ \;+\alpha_{1}sign(e_{ij}(t))\left|e_{ij}{\;}^{2\phi_{1}-1}(t)\right|+\alpha_{2}sign(e_{ij}(t))\left|e_{ij}{\;}^{2\phi_{2}-1}(t)\right|]\\ \;-n_{ij}(t)\}\text{=}-\gamma \left(\alpha_{0}e_{ij}^{2}(t)+\alpha_{1}\left|e_{ij}{\;}^{2\phi_{1}}(t)\right|+\alpha_{2}\left|e_{ij}{\;}^{2\phi_{2}}(t)\right|\right)\\ \;-n_{ij}(t)\times e_{ij}(t)\leq -2\alpha_{0}V(e_{ij}(t))-2\alpha_{1}V^{\phi_{1}}(e_{i,j}(t))\\ \;-2\alpha_{2}V^{\phi_{2}}(e_{ij}(t))-\sigma V(e_{ij}(t))=-2(\alpha_{0}+\frac{1}{2}\sigma )V(e_{ij}(t))\\ \;-2\alpha_{1}V^{\phi_{1}}(e_{ij}(t))-2\alpha_{2}V^{\phi_{2}}(e_{ij}(t)) \end{array}$$

According to Lemma 1, the convergence time of FTCRNN model (12) can be obtained.


(35) $$\begin{array}{l} T_{ij}\leq \frac{1}{\left(2\alpha_{0}+\sigma \right)\left(1-\phi_{1}\right)}\ln\;\left(1+\frac{2\alpha_{0}+\sigma }{2\alpha_{1}}\right)\\ \;+\frac{1}{\left(2\alpha_{0}+\sigma \right)\left(\phi_{2}-1\right)}\ln\;\left(1+\frac{2\alpha_{0}+\sigma }{2\alpha_{2}}\right) \end{array}$$

**Remark 1**. As observed in Equations (30, 34), the parameters (α_0_, α_1_, α_2_, ϕ_1_*, and* ϕ_2_) of NAF (1) are closely related to the convergent time of the FTCRNN model. Considering the above equations and the numerical simulation in the next section, we set α_0_ = 50, α_1_ = α_2_ = 0.2, ϕ_1_ = 1.3, ϕ_2_ = 0.2 to ensure the FTCRNN model achieve better convergence and robustness.

### Dynamic problems simulation verification

In this subsection, three experiments using the NAF-based FTCRNN model are demonstrated. In example 1, a third-order DSE (TODSE) is solved by using the proposed NAF-based FTCRNN model. The FTCRNN-based robotic manipulator trajectory tracking application is applied in example 2. In example 3, an application of electric circuit currents computation using the NAF-based FTCRNN model is presented.

#### Example 1. TODSE problem solving

In this subsection, the proposed FTCRNN model is used to solve a TODSE problem on the basis of the following dynamic coefficient matrices. Additionally, the ZNN model (9) activated by other three AFs in Table 6 is also applied to solve the same TODSE problem for comparison.


$$\begin{array}{l} A=\left(\begin{array}{ccc} \sin3t & \cos3t & \sin t\\ -\cos3t & \sin3t & \cos t\\ -\sin t & -\cos2t & \sin3t \end{array}\right)\\ B=\left(\begin{array}{ccc} 20 & 0 & 10\\ 0 & 30 & 15\\ 0 & 15 & 20 \end{array}\right)\\ C=\left(\begin{array}{ccc} \sin3t & \cos3t & \sin t\\ -\cos3t & \sin3t & \cos t\\ \sin t & \cos t & \sin3t \end{array}\right) \end{array}$$

**Figure 12:**
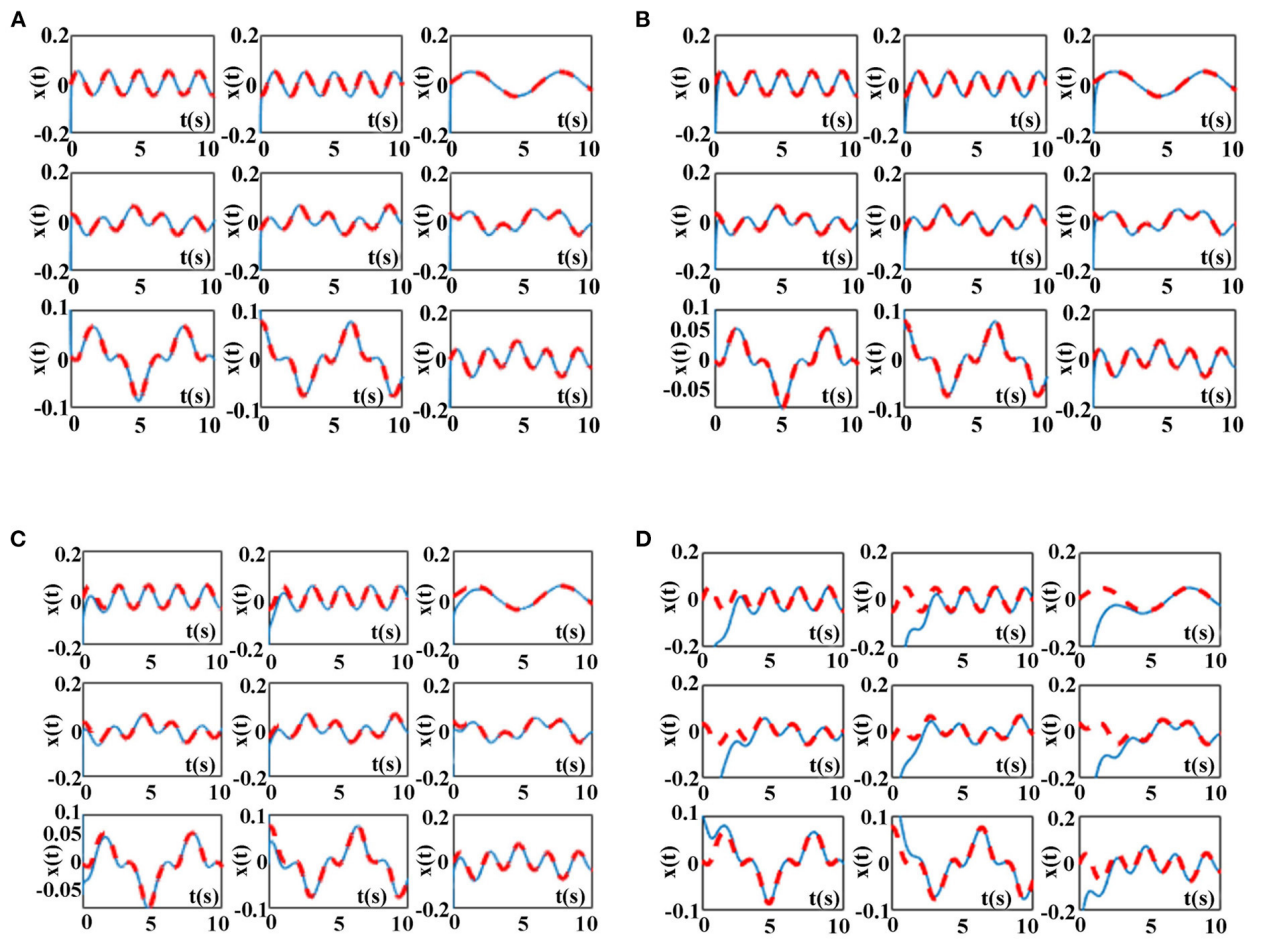
TODSE (5) solved by ZNN model (9) and the proposed NAF-based FTCRNN model (11) without noise. **(A)** Solved by FTCRNN model (11) without noise. **(B)** Solved by VAF-based ZNN model (9) without noise. **(C)** Solved by SBPAF-based ZNN model (9) without noise. **(D)** Solved by LAF-based ZNN model (9) without noise.

The simulation results of the ZNN model (9) activated by other three AFs in Table 6 and the proposed NAF-based FTCRNN model (11) for solving the above TODSE (5) in no noise environment are presented in Figure 12. The red dotted curves and blue solid curves are the theoretical solutions and neural state solutions of TODSE (5), respectively. As observe in Figure 12, the ZNN model (9) and the proposed NAF-based FTCRNN model all effectively solve the TODSE (5).

**Figure 13:**
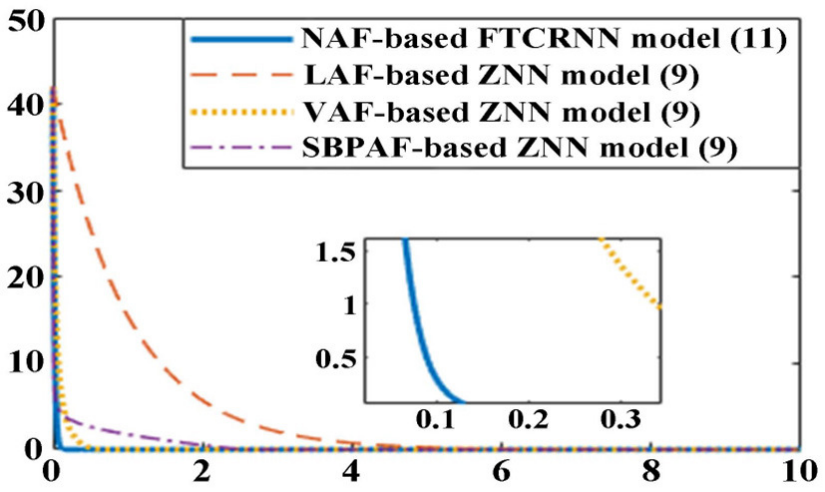
Residual errors of the ZNN model (9) and the NAF-based FTCRNN model (11) for solving TODSE without noise.

To clearly compare their effectiveness for solving TODSE (5) in no noise environment, the simulated residual errors ||*A*(*t*)*X*(*t*) − *X*(*t*)*B*(*t*) + *C*(*t*)||_*F*_ of the ZNN model (9) activated by other three AFs in Table 6 and the proposed NAF-based FTCRNN model are presented in Figure 13. It is obvious that all the models possess the ability to converge to the theoretical solution of TODSE (5), but their effectiveness are quite different. The proposed FTCRNN model (11) spends only about 0.15 s to find the theoretical solution of TODSE (5), which is the most effective candidate for solving TODSE in no noise environment.

Moreover, according to Equation (30), the convergent time of the FTCRNN model in no noise environment can be calculated.


(36) $$\begin{array}{l} T_{s}\leq \frac{1}{2\alpha_{0}\left(1-\phi_{1}\right)}\ln\;\left(1+\frac{\alpha_{0}}{\alpha_{1}}\right)+\frac{1}{2\alpha_{0}\left(\phi_{2}-1\right)}\ln\;\left(1+\frac{\alpha_{0}}{\alpha_{2}}\right)\\ \;\approx 0.7s \end{array}$$

The theoretical convergent time of the FTCRNN model in no noise environment is 0.7 s, and the simulated convergent time is about 0.15 s, which prove that the theoretical analysis result is consistent with the experimental result.

**Figure 14:**
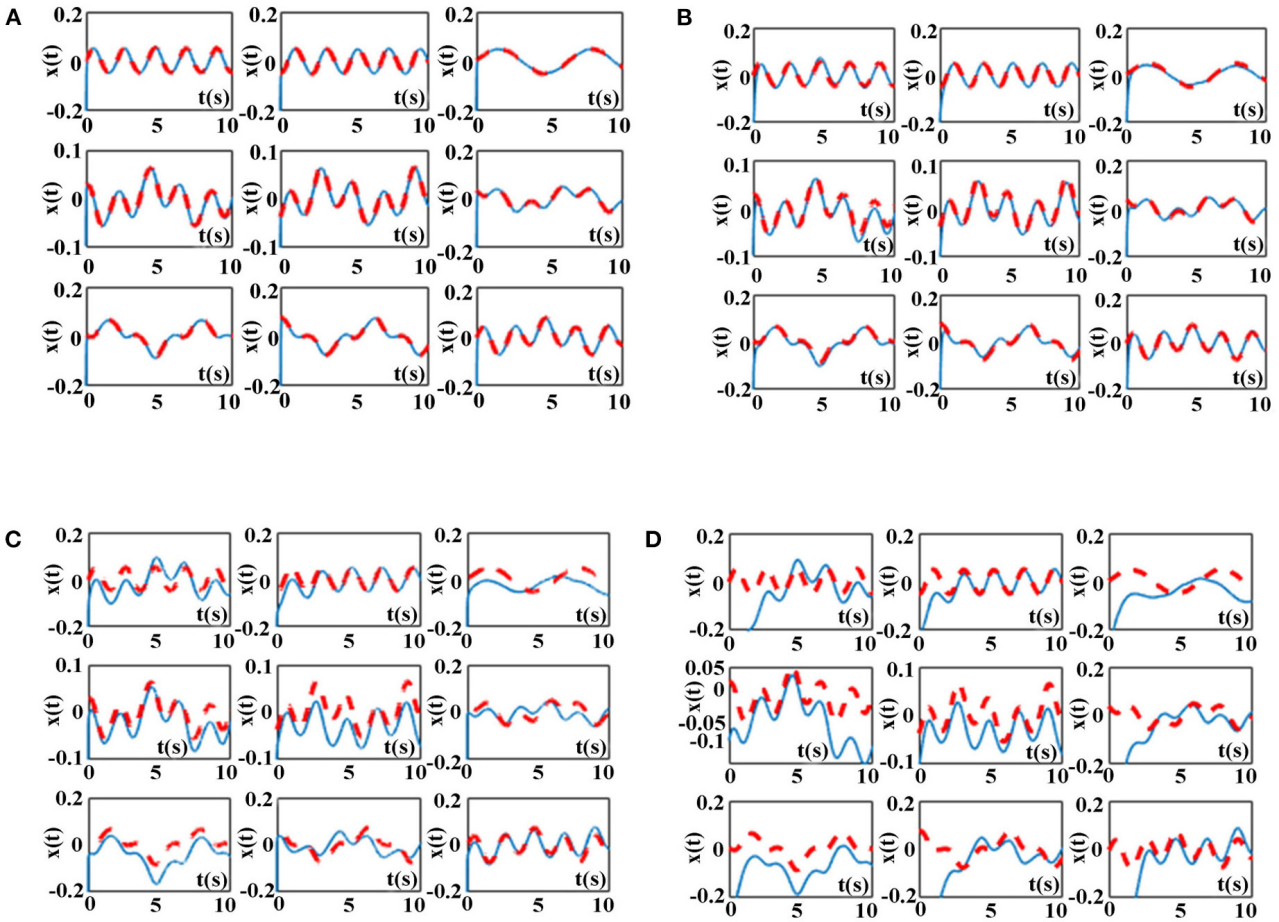
TODSE (5) solved by ZNN model (9) and the proposed NAF-based FTCRNN model (12) in noisy environment. **(A)** Solved by FTCRNN model (12) in noisy environment **(B)** Solved by VAF-based ZNN model (9) in noisy environment. **(C)** Solved by SBPAF-based ZNN model (9) in noisy environment **(D)** Solved by LAF-based ZNN model (9) in noisy environment.

**Figure 15:**
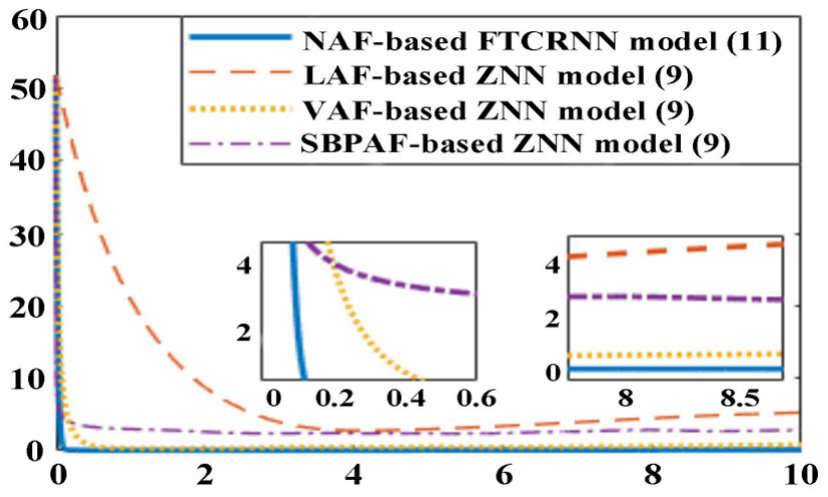
Residual errors of ZNN model (9) and NAF-based FTCRNN model for solving TODSE in noisy environment.

Considering the inevitable noises in practical application, ZNN model (9) activated by other three AFs in Table 6 and the proposed NAF-based FTCRNN model (12) are also applied to solve the TODSE (5) in noisy environment, and the dynamic mixed-noise matrix *N(t)* is presented as below. The corresponding simulation results for solving TODSE (5) in noise-polluted environment are presented in Figures 14, 15.


(37) $$\begin{array}{l} N\left(t\right)=\left(\begin{array}{ccc} 1.5\sin t & 0.5t & 1.5\\ 1.5e^{-t} & 0.5+0.1t & 1.5\cos t+0.1\\ 1.5\sin t+0.1t & 1.5\cos t+e^{-t} & e^{-t}+0.1 \end{array}\right) \end{array}$$

As shown in Figure 14, the proposed NAF-based FTCRNN model (12) and the VAF-based ZNN model (9) still effectively solve TODSE (5) in noisy environment, but the ZNN model (9) activated by the LAF and SBPAF fail to solve TODSE (5) due to the influence of noises.

The residual errors of the ZNN model (9) activated by other three AFs in Table 6 and the proposed FTCRNN model (12) are also presented in Figure 15 for further comparison. However, it can be seen from Figure 15 that the convergence performance of the FTCRNN model and the VAF-based ZNN model is completely different. With the increase of solution time, the residual error of the VAF-based ZNN model is increasing, while the residual error generated by the FTCRNN model remains stable at zero.

Moreover, according to Equation (34), the theoretical convergent time of the FTCRNN model in noisy environment can be calculated.


(38) $$\begin{array}{l} T_{s}\leq \frac{1}{\left(2\alpha_{0}+\sigma \right)\left(1-\phi_{1}\right)}\ln\;\left(1+\frac{2\alpha_{0}+\sigma }{2\alpha_{1}}\right)\\ \;+\frac{1}{\left(2\alpha_{0}+\sigma \right)\left(\phi_{2}-1\right)}\ln\;\left(1+\frac{2\alpha_{0}+\sigma }{2\alpha_{2}}\right)\approx 0.7s \end{array}$$

The theoretical convergent time of the FTCRNN model in noisy environment is 0.7 s, and the simulated convergent time is about 0.2 s, which further indicates the theoretical analysis result is consistent with the experimental result.

For showing the different performances of the four AFs based models, comprehensive comparisons are listed in Table 7. Obviously, the NAF-based FTCRNN model is the best one not only in convergence performance but also in robustness. Besides, only the NAF-based FTCRNN model realizes fast convergence under the condition of noise interference.

**Table 7:** Comparisons of the LAF, SBPAF, VAF, and NAF based models for solving TODSE.

	**LAF-based ZNN (9)**	**SBPAF-based ZNN (9)**	**VAF-based ZNN (9)**	**FTCRNN**
Convergence	Poor	Poor	Better	Best
Robustness	Weak	Weak	Strong	Very strong
Convergence time in ideal environment	5.5 s	2.5 s	0.6 s	0.15 s
Convergence time in noise environment	/	/	/	0.2 s

Based on the above analysis, we can conclude that the proposed FTCRNN model has better robustness and effectiveness than recently reported works.

**Remark 2**. According to the hypothesis in Theorem 2 and the noise matrix *N(t)*, we have |*n*_*ij*_(*t*)| ≤ σ|*e*_*ij*_(*t*)| and |*n*_*ij*_(*t*)| ≤ 2.5. We can also have |*e*_*ij*_(*t*)| ≈ 50 from Figure 15. Then, the value of parameter σ is σ ≥ 0.005, and we set σ = 0.07.

#### Example 2. Application to robotic manipulator

The research on robots becomes popular in recent years (Jin et al., 2020; Zhang et al., 2020a, 2021; Jin, 2021b). Therefore, the FTCRNN-based RM trajectory tracking application is realized to further verify the practical application feasibility of the FTCRNN model.

**Figure 16:**
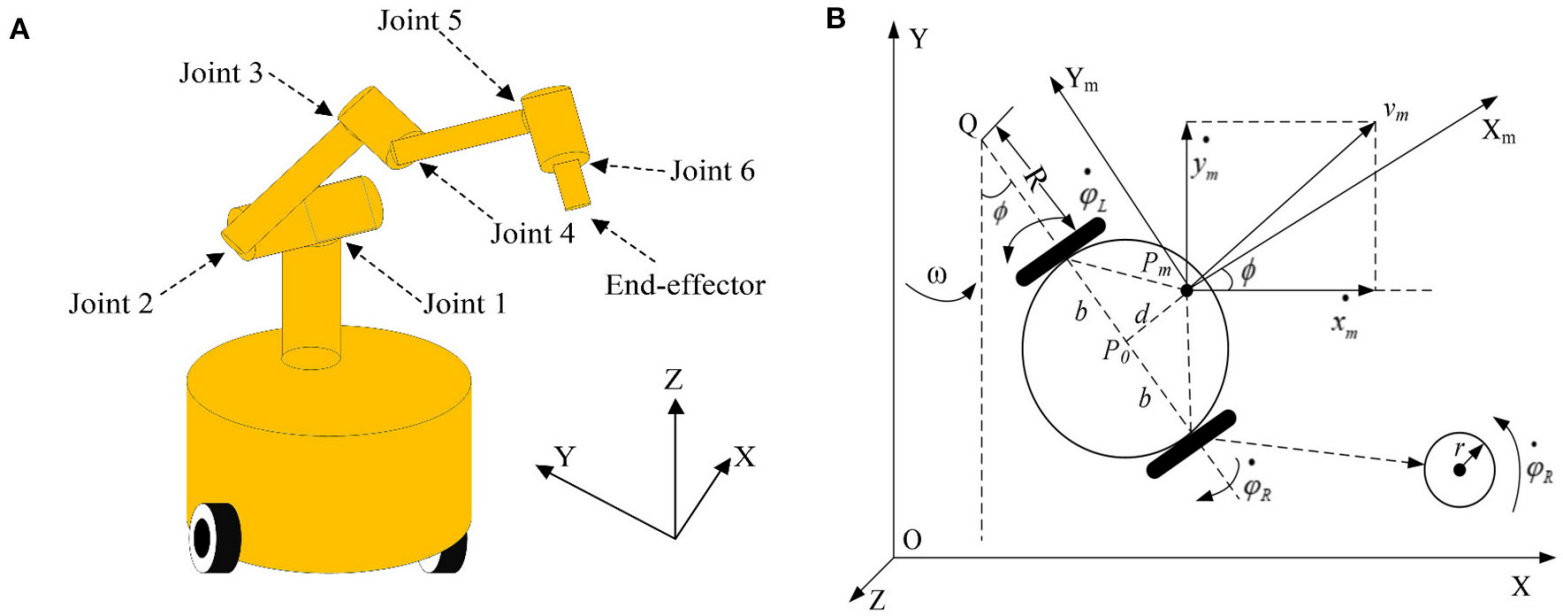
Modeling of the RM. **(A)** Three-dimensional structure of the RM. **(B)** Geometrical model of the RM.

The three-dimensional structure and geometrical model of a RM are presented in Figure 16. As observed in Figure 16A, the RM consists of a movable platform with a manipulator with six joints, and its geometrical model is depicted in Figure 16B.

Based on the modeling method in Xiao and Zhang (2014a) and Zhao et al. (2021), its kinematic matrix equation in velocity level is presented as follows.


(39) $$\begin{array}{l} {\overset{•}{r}}_{m}=B\left(\phi ,\theta \right)\overset{•}{\Theta } \end{array}$$

where *r*_*m*_ is an end-effector position vector, Θ = [φ^*T*^, θ^*T*^] ^*T*^ denotes a combined RM angle vector. Besides, ${\overset{•}{r}}_{m}\in {\mathbb{R}}^{m}$ and $\overset{•}{\Theta }$ are time derivatives of *r*_*m*_ and Θ, respectively. *B*(ϕ, θ) ∈ ℝ^*m*×(2+*n*)^ is defined as


(40) $$\begin{array}{l} B\left(\phi ,\theta \right)=\left[\begin{array}{cc} M & 0\\ 0 & 0 \end{array}\right]+J\left(\phi ,\theta \right)\left[\begin{array}{cc} N & 0\\ 0 & I \end{array}\right] \end{array}$$

where


(41) $$\begin{array}{l} M=\frac{r}{2}\left[\begin{array}{cc} \cos\phi & -\sin\phi \\ \sin\phi & \cos\phi \end{array}\right]\left[\begin{array}{cc} 1 & 1\\ -d/b & d/b \end{array}\right],N=\frac{r}{2\text{b}}{\left[\begin{array}{c} -1\\ 1 \end{array}\right]}^{T} \end{array}$$

and *J* (φ, θ) is the Jacobian matrix.

Here, $r_{md}\left(t\right)\in {\mathbb{R}}^{m}$ is the desired path to be tracked.*r*_*m*_(*t*) is the actual path of the RM end-effector, and we have


(42) $$\begin{array}{l} f\left(\Theta ,t\right)=r_{m}\left(t\right)\to r_{md}\left(t\right) \end{array}$$

where *f* (•) stands for a continuous non-linear kinematic map of the RM.

Then, differentiating Equation (37) yields the following velocity level kinematic equation.


(43) $$\begin{array}{l} B\left(\phi ,\theta \right)\overset{•}{\Theta }={\overset{•}{r}}_{m}\left(t\right)\to {\overset{•}{r}}_{md}\left(t\right) \end{array}$$

On the basis of the above discussion, it is clear that the desired path $r_{md}\left(t\right)\in {\mathbb{R}}^{m}$ is given in advance, and the joint and wheel trajectories of the RM are required to be calculated by the RNN models. Therefore, the RNN models for solving the above tracking task are constructed as the following steps.

Firstly, a vector-valued error matrix is defined.


(44) $$\begin{array}{l} E\left(t\right)=r_{md}\left(t\right)-r_{m}\left(t\right) \end{array}$$

The trajectory tracking task is transformed to ensure each element *e*_*i*_(*t*) of *E*(*t*) converging to 0, and the following formula is used to guarantee its convergence.


(45) $$\begin{array}{l} \overset{•}{E}\left(t\right)=-\lambda \text{Ψ}\left(E\left(t\right)\right) \end{array}$$

For better comparison, both of the FTCRNN model (12) and the SBPAF-based ZNN model (9) are applied to ensure the convergence of *E(t)* in noisy environment, and the corresponding models are shown as follows.


(46) $$\begin{array}{l} B\overset{•}{\Theta }={\overset{•}{r}}_{md}\left(t\right)+\lambda {\text{Ψ}}_{1}\left(r_{md}\left(t\right)-r_{m}\left(t\right)\right)+N\left(t\right) \end{array}$$

(47) $$\begin{array}{l} B\overset{•}{\Theta }={\overset{•}{r}}_{md}\left(t\right)+\lambda {\text{Ψ}}_{2}\left(r_{md}\left(t\right)-r_{m}\left(t\right)\right)+N\left(t\right) \end{array}$$

where ψ_1_(•) is the proposed NAF in Equation (1), and ψ_2_(•) is the SBPAF in Table 6.

**Figure 17:**
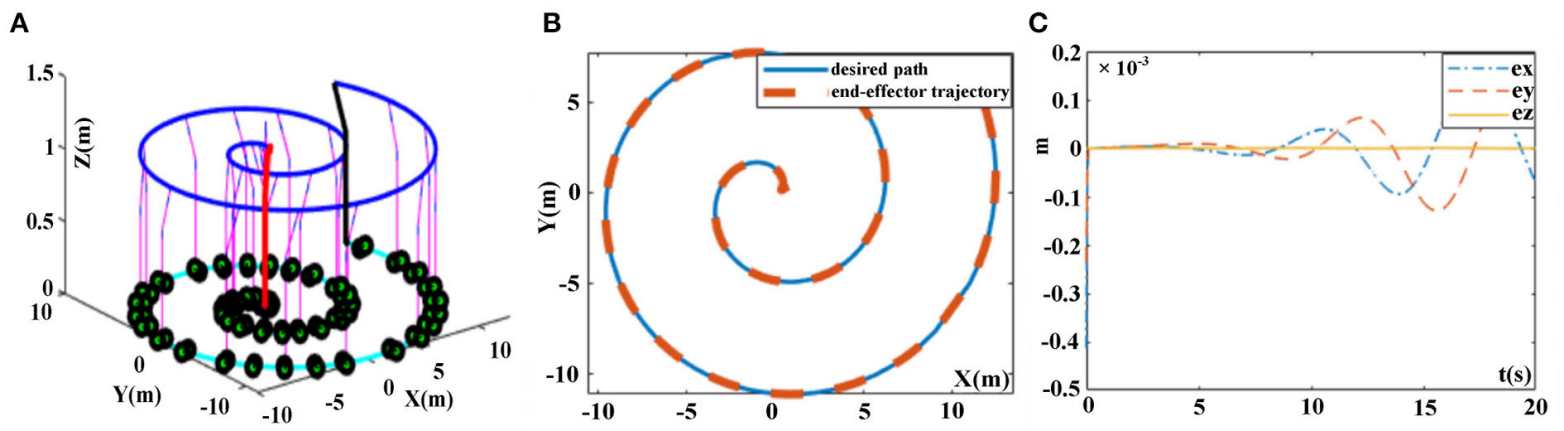
Trajectory tracking results synthesized by the FTCRNN-based model (41) in noisy environment. **(A)** Whole tracking motion trajectories. **(B)** Desired path and actual trajectory. **(C)** Tracking errors.

**Figure 18:**
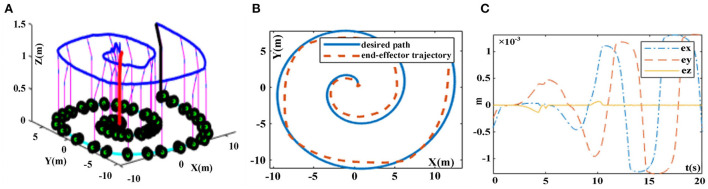
Trajectory tracking results synthesized by the ZNN-based model (42) in noisy environment. **(A)** Whole tracking motion trajectories. **(B)** Desired path and actual trajectory. **(C)** Tracking errors.

The desired tracking path is a helical line, and the initial state of the robotic manipulator is set as Θ *(0)* = *[*π*/6*, π*/3*, π*/6*, π*/3*, π*/3*, π*/3]*^*T*^, and *N(t)* = *0.025*cos*t*. The trajectory tracking experiment results of robotic manipulator synthesized by the proposed FTCRNN-based model (41) and the SBPAF-based ZNN model (42) are displayed in Figures 17, 18.

It can be seen from Figures 17, 18 that the FTCRNN-based model (41) successfully completes the tracking task in noisy environment, and the end-effector trajectory coincides with the desired path. Besides, the tracking errors of the FTCRNN-based model (41) are all <0.1 cm. However, the SBPAF-based ZNN model (42) fails to complete the tracking task due to the disturbance of additive noises, and tracking errors of the SBPAF-based ZNN model (42) are all more than 1.5 cm. Furthermore, compared with the RM trajectory tracking methods in Xiao and Zhang (2014a) and Zhao et al. (2021), the most significant improvement of this work lies in its strong robustness to noises, which ensures the success of actual RM trajectory tracking application considering noises in reality.

## Conclusion

In this paper, a NAF is proposed and employed in different RNN models to handle sentiment classification problem and dynamic problems solving. Experiment results of IMDB sentiment classification demonstrate that the NAF-based RNN models have better training and test accuracy, lower and more stable loss values than other AFs in Table 1 based RNN models. Besides, based on the NAF, the FTCRNN model is developed. The effectiveness and robustness of the proposed FTCRNN model are analyzed in theory and simulation experiment. Moreover, the FTCRNN-based robotic manipulator trajectory tracking application is carried out to verify the practical application feasibility of the FTCRNN model. Furthermore, the application of electric circuit currents computation further verifies the wide applicability of the FTCRNN model. This work provides a promising choice for the selection of AF of RNN models in sentiment classification and time-varying problems solving applications. The fixed-time convergence and strong robustness of the proposed NAF-based FTCRNN model guarantee its real-time online computing capability in noisy environment. Note that, only sentiment classification and time-varying problems solving of the NAF-based RNN models are considered in this paper. Thus, extending the applications of the NAF in different RNN models for text classification and translation needs future research.

## Data availability statement

The original contributions presented in the study are included in the article, further inquiries can be directed to the corresponding author.

## Author contributions

All authors listed have made a substantial, direct, and intellectual contribution to the work and approved it for publication.

## Funding

This work was supported by the Guiding Plan Project of Scientific and Technological Innovation and Development in Changde (Grant/Award Number: 2020ZD37) and the Scientific Research Project from Hunan University of Arts and Science (Grant/Award Number: 21ZD03).

## Conflict of interest

The authors declare that the research was conducted in the absence of any commercial or financial relationships that could be construed as a potential conflict of interest.

## Publisher's note

All claims expressed in this article are solely those of the authors and do not necessarily represent those of their affiliated organizations, or those of the publisher, the editors and the reviewers. Any product that may be evaluated in this article, or claim that may be made by its manufacturer, is not guaranteed or endorsed by the publisher.
